# Beyond the Complexity, Accuracy, and Fluency Framework: A Meaning-Oriented Approach to Cognitive and Affective Influences on L3 Language Production

**DOI:** 10.3390/brainsci16070722

**Published:** 2026-07-06

**Authors:** Fuqiang Ran, Jeroen van de Weijer, Zhiyuan Zheng

**Affiliations:** 1 College of International Studies, Shenzhen University, No. 3688, Nanhai Avenue, Nanshan District, Shenzhen 518060, China; 2500221020@mails.szu.edu.cn; 2 School of Foreign Studies, China University of Petroleum, East China, No. 66, Changjiang West Road, Huangdao District, Qingdao 266580, China; z25170066@s.upc.edu.cn

**Keywords:** working memory capacity, language anxiety, language proficiency, language production, third language acquisition

## Abstract

**Highlights:**

**What are the main findings?**

- The main effect of language proficiency is significant.
- The interaction between working memory capacity (WMC) and language proficiency is associated with language production.

**What are the implications of the main findings?**

- Language proficiency plays a crucial role in language production.
- WMC may contribute to language production, and its effect on German L3 language production in Cantonese (L1)–English (L2) speakers is mostly moderated by language proficiency.

**Abstract:**

**Background/Objectives**: Previous research has investigated the impact of cognitive as well as emotional predictors, including WMC and language anxiety on language production. These studies often adopt the Complexity, Accuracy and Fluency (CAF) framework to assess language production. However, outcomes on language production related to meaning have played an underrepresented role in this model, resulting in previous findings that were inconsistent. **Methods**: The present study addresses this limitation by integrating the meaning-oriented Communication Content Function (CCF) model, based on Systemic Functional Linguistics, into the CAF framework, resulting in a revised model for language production evaluation under experimental conditions, which we call the Formal and Functional Evaluation (FFE) model. This model allows for a more comprehensive investigation of the impact of WMC and language anxiety on language production. Using data from L3 acquisition of German by L1 Cantonese and L2 English speakers, we examine how WMC, language anxiety, and language proficiency interact in shaping production outcomes. **Results**: Multiple regression analyses show that the main effects of WMC and language anxiety are not significant in most language production outcomes, whereas the main effect of language proficiency is significant in almost all outcomes. Also, a significant interaction between WMC × language proficiency is observed, although language anxiety × language proficiency as well as language anxiety × WMC are not. **Conclusions**: These results suggest that cognitive and affective factors influence language production primarily through interaction effects moderated by language proficiency.

## 1. Introduction

The ultimate goal of learning a foreign language is to talk with others. Previous studies have uncovered a number of factors that influence language production. Generally, affective factors, like language anxiety, as well as cognitive constructs, including WMC and language performance, are relevant factors [1,2,3,4]. The interaction of such factors has received some attention but remains underexplored on the basis of particular case studies. This study carries out a regression analysis on the predictive power of WMC, language anxiety, language performance and their interaction in L3 German language production. The results lead us to propose a revision to the widely used Complexity, Accuracy and Fluency model for L3 language production [5].

The influence of language anxiety on cognitive performance in general has been a topic of research at least since 1978 [6]. A number of studies have focused on the correlation between language anxiety and language production in a foreign language [7,8,9,10,11]. Most of these studies report a positive correlation between language anxiety and language production, i.e., greater language anxiety may lead to a decline in fluency as well as accuracy of language production [7,12]. However, some studies found a negative correlation between these factors, i.e., speakers with higher language anxiety tend to have better language production [10]. Trebits found that language anxiety may improve language production because more effort is elicited in the process stage to produce more complex words and sentences [10]. Moreover, there are also some studies that failed to find a significant relationship [13]. One possible reason for these contradictory findings might be that the outcomes used to evaluate language production in previous studies are different. Early research focused on a single dimension for language production evaluation, but later studies took more dimensions into account, and recent studies take almost all dimensions of L2 Complexity, Accuracy and Fluency (henceforth CAF; see [5]) into consideration. CAF is an L2 production evaluation model proposed by Skehan in 1998 that has been widely adopted in production studies. However, CAF mostly measures the formal features of language and does not take into consideration how well meaning is conveyed in an L2. Previous studies have therefore tended to neglect this aspect of foreign language production. In order to fill this gap, the present study intends to incorporate an evaluation framework by taking the approach to meaning advocated in Systemic Functional Linguistics (SFL) into account [14]. This model is called the Communication Content Function (henceforth: CCF) model. The CAF model, based on Levelt’s work [15], as well as the new CCF framework, are grounded in the cognitive mechanism of language production. This mechanism applies to both L1 and L2/L3. Although L2 and L3 may be influenced differently by L1 transfer effects, we tentatively assume that the main factors underlying L2 and L3 production are sufficiently similar to use both in L3 language production research [5,7,9].

Language anxiety is treated as the affective factor examined in this study, whereas WMC is treated as the cognitive factor. The correlation of WMC with language production is a topic that has produced considerable research [16,17,18,19,20]. Its role can be accommodated by Levelt’s speech production theory [15]. Previous studies on this topic consider WMC to be an important component in the conceptualizing stage of language, which forms the starting point of speech production [15,21]. Many studies have investigated the relation between WMC and language production. Some of these report a positive correlation between WMC and language production quality [19,22,23], while others find no significant effect [24]. There is a number of potential reasons for these contradictory findings. The first is that the predictive value of WMC on language production might be too small to find [25] or be influenced by other factors [17,25]. Secondly, differences in evaluation method may also play a role. The last potential reason is that WMC may not yield a main effect on language production. Soilemezidi et al. discovered that working memory is crucially tied to process speed to predict verb-related production [24,26]. Other studies, too, report limited influence of WMC [20]. Sun et al. conclude that the influence of WMC is only significant among high-proficiency speakers. The reason may be that high-proficiency speakers have adequate linguistic resources that they can retrieve: the relatively high WMC of such speakers may help them to retrieve linguistic resources more efficiently [17].

Language proficiency is also an important factor for language production. For example, Bulté and Roothooft find that many linguistic outcomes differ significantly across speakers of different proficiency levels [27]. Bao finds the same for the specific issue of phrasal complexity [28]. The reason for this may be related to the processes of lexical retrieval and syntactic construction, which are relatively more highly automated for higher-proficiency speakers [28,29]. For this reason, language proficiency is also included in this study as a predictor so as to investigate its main effect and interaction effect with language anxiety and WMC.

The interaction among WMC, language proficiency and language anxiety was recently investigated for second language reading, but there are not many studies on L2/L3 language production [30]. The present study thus performs a multiple regression analysis to re-investigate the predictive power of WMC and language anxiety and how their statistical relationship with language production is moderated by language proficiency. Based on previous studies, this study puts forward two hypotheses:

**Hypothesis 1:**  *Language proficiency is associated with language production outcomes, but WMC and language anxiety do not predict language production independently of language proficiency.*

**Hypothesis 2:**  *Language proficiency moderates the effects of working memory capacity and language anxiety on language production.*

To summarize, this study seeks to construct a more comprehensive evaluation framework on L3 language production proficiency by investigating the different predictors involved in L3 language production, including language anxiety, language proficiency and WMC, in a multiple regression analysis. To highlight the increased role of meaning-related outcomes, we call this model the Formal and Functional Evaluation (FFE) model, which takes both formal and meaning outcomes into consideration. It consists of both the CAF measures and the selected CCF measures, which ensures that not only the formal linguistic performance but also meaning conveyance is taken into consideration.

## 2. Materials and Methods

### 2.1. Participants, Tasks and Data Collection

The data in this study came from an open database, the L3HK Repository [31] (the protocol of the L3HK Repository was approved by the Joint Chinese University of Hong Kong—New Territories East Cluster Clinical Research Ethics Committee). This database includes data from 906 speakers with Cantonese as L1 and English as L2. Subjects in this study were those who spoke German as their L3, because the number of German L3 speakers was relatively high and their gender distribution as well as their age range were the most balanced [31]. The current study therefore filtered the original database, with filters for “German” in L3 and “Available” for WMC, leading to a final set of statistics from 91 subjects. They were recruited in the college, with 64 female and 27 male speakers, aged between 18 and 25. These speakers were asked to recount the story of the wordless picture book “Frogs, where are you?” in German, and the storytelling was audio-recorded.

### 2.2. Predictor Assessments

Speakers completed two questionnaires to assess their proficiency level as well as degree of language anxiety [32,33]. The language anxiety data was collected by a classical questionnaire, the Modern Language Learner Questionnaire. This questionnaire collected lots of statistics of language learning, and language anxiety is an important part. This questionnaire asked subjects to judge whether they agree or disagree with a number of statements on a six-point Likert scale in Section 1 and required them to answer questions on a six-point Likert scale ranging from “not at all” to “very much” in Section 2. The language anxiety score was formed by adding up the related items (Cronbach‘s α = 0.78) [32]. As for the language proficiency questionnaire, subjects were asked to report their linguistic ability in different settings, across listening, reading, speaking and writing, on a Likert scale from 1–5. The language proficiency questionnaire was validated by previous research [32,33]. MacIntyre et al. conducted an experimental study in which they adopted questionnaires to collect self-report language proficiency data and tasks in reading, listening, writing and speaking to collect actual proficiency data. In their results, self-reported language proficiency was highly correlated with actual proficiency at correlates ranging between 0.51 and 0.72 [33]. As for the working memory statistics in this database, only 293 subjects chose to complete the optional running working memory task [34]. Subjects would get one point if they could recall each item in the correct position after hearing 3–7 letters. The percentage of correct recalls was calculated to be the WMC statistic [34]. Thus, the present study did not include all L3HK subjects because not all of them completed the WMC task.

### 2.3. Outcomes, Data Coding and Analysis

The present study not only used the traditional CAF measures to investigate language production performance, but also took meaning-related measures, namely the CCF measures, into consideration to evaluate how effectively meaning was conveyed in the subjects’ language production [5,14]. We refer to this integrated model as the Formal and Functional Evaluation (FFE) model. It consists of all formal measures that are recognized in CAF (see Section 2.3.1) and the meaning-related measures in CCF (see Section 2.3.2). In this case, the FFE model has high reliability on L3 language production evaluation. The FFE is formed by directly adding up CAF and CCF because they reflect two different aspects of language production. The addition of raw data can reflect the observed experimental distribution without integrating a new distribution hypothesis.

#### 2.3.1. CAF Model Section

The CAF outcomes express the Complexity, Accuracy and Fluency of subjects’ oral performance. Table 1 shows all CAF section outcomes of the FFE model used in this study.

The moving average type-token ratio (MATTR) is considered to be a unique measure among lexical diversity (LD) measures because it can be used to standardize the LD statistics in different text volumes so as to avoid different text size effects [35,36]. Mean Dependency Distance (MDD) and Embedding Depth (ED) are two syntactic complexity outcomes. MDD refers to the average location distance between two semantically related words, and ED measures the degree of syntactic embedding within a sentence [37,38]. These two outcomes not only reflect syntactic richness but also the processing load in language production, which is closely related to cognitive functions like working memory [37,38]. The reason why they are treated separately is that they measure syntactic complexity from different perspectives. ED measures the depth of embedding in syntactic structure, while MDD measures the linear distance of syntactic dependency—depth of embedding does not ensure longer distance [37]. In this study, all three outcomes are calculated using the R statistical language (R 4.6.1) [39]. To be specific, MDD and ED were calculated with the help of the udpipe package (1.0) [40] and the German GSD UD model (version 2.5), while MATTR was calculated within the R general package.

Repetition proportion and retrace proportion are included as fluency outcomes because they are typical disfluency phenomena. Similarly, word errors and utterance errors are measured to reflect accuracy. These outcomes were annotated by two raters, and any discrepancy was solved by a final discussion between these raters. As for the computation, the counts of word errors and utterance errors were divided by the number of words and utterances to obtain their proportion, and the number of repetitions and retracing were divided by the number of words to obtain their proportion statistics. All these outcomes were extracted from the database. Accuracy, fluency and their metric outcomes were negated for the lower-is-better feature [31].

The CAF statistics were calculated using Formula (1). The outliers were detected by Interquartile Range (IQR), where we took a 1.5 IQR as the multiplier. Outliers are defined as those lower than Q1-1.5 × IQR or higher than Q3-1.5 × IQR [41]. The results show that all outcomes have outliers. MDD and ED are standardized by fractional rank normalization following Formula (2) because the MDD and ED outcomes can be influenced by sentence length; the numeric proportion does not need to be preserved. Also, it does not have natural boundaries, and almost 5% of the data here are outliers. The outcomes for MATTR, repetition proportion, retracing proportion, word error proportion and utterance proportion were normalized using the min–max rescaling given their boundness and natural range between 0 and 1. These proportions reflect a genuine difference, and their outliers genuinely occur. Moreover, these proportions can be used for comparison: the statistical difference should make it possible to examine the magnitude of each dimension’s contribution [42].

(1) $$\begin{array}{c} \mathrm{Complexity}=\mathrm{MDD}+\mathrm{ED}+\mathrm{MATTR}\\ \mathrm{Fluency}=\mathrm{Repetition}\;\mathrm{proportion}+\mathrm{Retracing}\;\mathrm{proportion}\\ \mathrm{Accuracy}=\mathrm{Word}\;\mathrm{error}\;\mathrm{proportion}+\mathrm{Utterance}\;\mathrm{error}\;\mathrm{proportion}\\ \mathrm{CAF}=\mathrm{Complexity}+\mathrm{Fluency}+\mathrm{Accuracy} \end{array}$$

(2) $$s_{i}=\frac{r_{i}-1}{n-1}$$

S_i_ = the normalized score of the i-th observation, ranging from 0 to 1;

r_i_ = the rank of the i-th observation in ascending order (1 = smallest, n = largest);

n = the total number of observations in the sample.

#### 2.3.2. CCF Model Section

In addition to the traditional CAF outcomes, this study proposes to incorporate meaning-related outcomes, consisted of several CCF model outcomes, into the FFE model so as to reflect how well subjects do in meaning conveyance.

Table 2 shows the version of the CCF model adopted in this study. The CCF model is put forward based on the Systemic Functional Linguistics (SFL) perspective [14]. Different from traditional grammar, SFL tends to assign more attention to the meaning of sentences. Since different kinds of meaning (literal, pragmatic and social) are of central importance in language, this factor should be included. Sentence structure is a typical example. Unlike traditional grammar, which places the emphasis on location and word class, SFL focuses on the role constituents play in the construction of meaning in a sentence. These kinds of meaning are referred to as textual and ideational meaning [43]. Ideational meaning consists of overall theme, core components and background. Overall theme refers to how many times the theme of the story appears. Core components evaluate whether the key plot of the whole story is narrated, while background consists of utterances before the starting point of the story [43]. Textual meaning has three sub-components, including temporality, reference and connectivity. Temporality and reference evaluate the accuracy of tense and reference words, while connectivity is related to the usage of connective words [43].

The present study does not include the interpersonal function in the newly put forward FFE model, instead only taking the ideational function and textual function of meaning into account. The reason is that the interpersonal function is not the focus of the narrative task. The interpersonal outcomes in the CCF model are designed to evaluate speakers’ engagement of listeners [14]. However, subjects in the present, narrative task were not asked to tell the story to someone but told the story in front of a microphone [31]. We therefore excluded interpersonal outcomes from the analysis. At the same time, how the story is narrated and whether the story is fully narrated relate to the experiment’s task achievement. This study therefore included the ideational function and textual function in the analysis framework.

CCF outcomes were collected by annotation, and the ideational, textual and total CCF statistic were calculated. Two raters were asked to conduct a blind rating. The rating handbook is presented in Appendix A. Both raters were postgraduate students in linguistics with sufficient linguistic background and basic German language proficiency. Before the formal rating procedure, ten percent of the samples were rated to establish the rating criteria.

After the rating procedure, a two-way mixed-effects model for consistency based on average ratings (ICC(3,k)) was used to test inter-rater reliability [44]. Table 3 shows that there was a high inter-rater reliability with respect to connectivity and background (ICC > 0.6) [45].

However, overall theme, core components, temporality and reference were excluded from further analysis because their ICCs were not higher than 0.6 [45].

The ideational, textual and CCF outcomes were calculated by Formula (3). Outliers for background and connectivity were detected based on the detection method mentioned above. These outcomes should be normalized by fractional rank normalization because they are exaggerated by subject. That is to say, background and connectivity were added up by annotated components since some subjects produced a large number of meaningless background and connectivity markers. For this reason, it is reasonable to apply fractional rank normalization.

CCF = Ideational(Background) + Textual(Connectivity)(3)

### 2.4. Data Analysis

This study adopts Multiple Regression (MR) for data analysis and all outcomes are included in the MR analysis. Table 4 illustrates the mean value and standard deviation of the different predictors and outcomes. A total of 91 groups of statistics are included here. As for the correlation, their relevant correlation coefficients are normally under 0.8, as Table 4 shows. Although there are high correlations (r > 0.8) among certain pairs of statistics, these are calculated directly from the component outcomes (see Table 5 below).

The major benefit of MR is that it can conduct a comprehensive investigation of the predictive value of WMC, L3 proficiency and L3 anxiety. The importance of these three factors can be seen by their R^2^ (see Table 6 below). WMC is treated as a continuous predictor to preserve crucial parts of the statistical data. To assess language production, both the CCF and the CAF model were adopted in this study. The CAF model has been widely used in production assessment, since it provides a comprehensive evaluation of lexical resources, grammatical accuracy and fluency. However, these outcomes do not include the semantic aspects of language. As pointed out above, the CCF model is intended to compensate for this. In this study, the CCF and CAF statistics will be treated as outcomes and WMC, L3 proficiency and L3 anxiety as predictors, to evaluate which model provides better insights into the relations between language anxiety, WMC and L3 language proficiency. Four regression models were constructed in this study. The first model is “language proficiency + WMC + language anxiety”, which investigates the predictive effects of language proficiency, WMC and language anxiety, respectively. The other three models are “WMC × language proficiency + language anxiety”, “language anxiety × language proficiency + WMC” and “language anxiety × WMC + language proficiency”, which map the interaction effect of “WMC × language proficiency”, “language anxiety × language proficiency” as well as “language anxiety × WMC”. As for the homogeneity of variance, this study adopts HC1 to correct the heteroscedasticity-consistent standard errors to all outcomes to account for potential heteroscedasticity [46]. Moreover, all outcomes are independent and show a linear relationship without multicollinearity. These ensure the applicability of MR (VIF results in Table A1).

Benjamini–Hochberg correction was adopted to correct the FDR multiple comparisons [47]. In order to investigate the interaction effects, this study conducted a post hoc interaction test on the “WMC x language proficiency + language anxiety” model. Also, the family-wise error rate (FWER) across the interaction effect of “WMC × language proficiency”, “language anxiety × language proficiency” and “language anxiety × WMC” was controlled by Holm’s sequential Bonferroni correction, and the corrected *p*-value < 0.05 is considered statistically significant [48]. The other models, including “language anxiety x language proficiency + WMC”, and “language anxiety × WMC + language proficiency”, were not subjected to further interaction analysis because no significant interaction can be witnessed (see Table 7). A post hoc interaction test was conducted in which the statistics were grouped by quartiles and the coefficients of WMC or language anxiety were calculated at each of these levels. Also, all materials can be accessed through the Supplementary Material link.

## 3. Results

MR was conducted to examine the relationships among the predictors and the continuous outcomes in this study.

Table 6 illustrates the overall adjusted R^2^ of the regression model. It can be seen that all four regression models explain a significant proportion of variance to predict the language production quality. Similarly, the models on the CCF and CAF scores both reach significance.

The accuracy, word error and utterance error outcomes are significant in all four models at a high adjusted R^2^. On the contrary, the complexity outcomes, retracing and fluency fail to reach significance in any model. It is also noteworthy that repetition is only significant in the “WMC × language proficiency + language anxiety” model.

Table 7 shows the coefficients of WMC, language proficiency and language anxiety in all four models. In general, language proficiency is the most frequently significant predictor across outcomes. As for language proficiency, it is associated with almost all outcomes. On the contrary, WMC, language anxiety, language anxiety × language proficiency and WMC × language anxiety are not significant in any outcomes, except that WMC are language anxiety is significant in repetition. In this case, the post hoc interaction test of the “language anxiety × language proficiency” and “WMC × language anxiety” were not analyzed in further detail. It is also noteworthy that WMC × language proficiency is associated with repetition.

**Figure 1:**
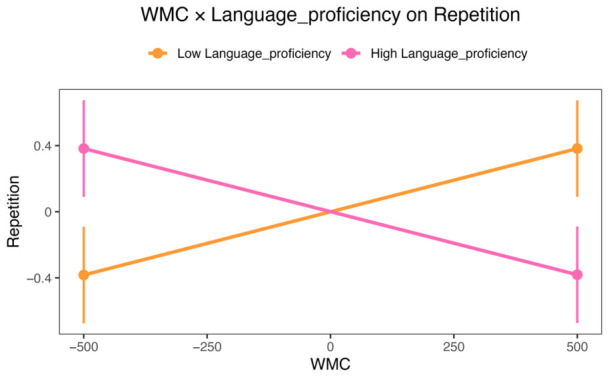
WMC × language proficiency interaction.

Table 8 illustrates the post hoc interaction test result of the interaction between WMC and language proficiency. It is evident that only the repetition outcome in high proficiency is significant. Moreover, the cross point can be witnessed in the interaction plots, as shown in Figure 1.

## 4. Discussion

As for the main effects of language proficiency, language anxiety and WMC, this study only corroborated the main effect of language proficiency on L3 language production, while the main effect of WMC and language anxiety reaches statistical significance only for the repetition outcome. Language proficiency is a significant predictor of language production, in line with previous findings [27,28]. This can be explained by Levelt’s speech production model [15]. This model divides speech production into six stages, including Concept, Lexical Selection, Syntactic Framing, Phonological Encoding, Articulation and Monitoring. Strong language proficiency ensures more reliable Lexical Selection, Syntactic Framing and Phonological Encoding [15]. Speakers with higher language proficiency have more linguistic resources available for language production.

On the other hand, WMC and language anxiety do not predict most language production outcomes. That is to say, WMC and language anxiety have limited predictive power with respect to language production. This finding contradicts previous findings, which report the significance of WMC and language anxiety on accuracy, while the significant main effect of WMC and language anxiety on repetition is line with previous studies [7,12,19,22]. Moreover, our finding is in line with several findings that do not support the main effects of WMC and language anxiety [13,24]. Many reasons may underlie this. The first is that FFE is affected by many factors and the influence of WMC is necessarily limited. Many predictors are involved, such as emotion, motivation, strategy and so on [17,25]. The second reason is that the influence sizes of WMC and language proficiency vary for speakers with different levels of language proficiency. WMC and language anxiety are significant for high-proficiency speakers’ fluency but not for low-proficiency speakers’ syntactic complexity. WMC and language anxiety therefore are not associated with the quality of language production across proficiency levels [20]. For these reasons, the significance of WMC and language anxiety is relatively weak, which explains the contradictory results found in the literature so far.

With respect to the second hypothesis, this study investigated the moderation effect of language proficiency with WMC and language anxiety on language production. It was found that only the interaction effect of WMC and language proficiency is significant but others are not significant, and the interaction effect is not significant in meaning measures.

This study found that the interaction between WMC and language proficiency may be associated with the repetition of L3 learners at low proficiency level.

As for the prediction for repetition, the interaction of WMC and language proficiency has a significant predictive effect on speaker’s L3 fluency. This can also be explained by Levelt’s speech production theory. In his theory, speech is produced through Concept, Lexical Selection, Syntactic Framing and Phonological Encoding stages. WMC, as an important cognitive function, plays an important role at the Concept stage, which has an effect on speakers’ meaning construction. This can be explained by Hasselhorn et al.’s Relay Race Model, in which phonological working memory helps speakers to construct simple concepts that are used to create final concepts for language production [49]. In this case, speakers with higher WMC do better in the Concept stage, because higher WMC ensures that they have logical and reasonable sentences to produce. However, lower language proficiency speakers with high WMC may face the dilemma that they have logical and reasonable sentences to say but their language competence is too limited to express them, arguably resulting in frequent repetitions.

Moreover, this study failed to find an interaction effect of language proficiency and language anxiety. This may be due to limitations with respect to the subjects in the Repository. The subjects in the Repository were college students who might rank highly with respect to attention capability development [50]. This may affect the predictive effect of language anxiety on language production [11]. Similarly, this study failed to find an interaction effect of language anxiety and WMC. The interaction effect of language anxiety and WMC is found to be moderate, but its generalizability is still unclear. Subjects in this study were mainly well-educated young people, and effects on other groups of speakers may be different [50].

It is also noteworthy to observe that accuracy and its metrics outcomes can be predicted in all models, but other outcomes in the CAF model cannot be predicted. This may be because accuracy is less likely to be affected by other factors and it is directly related to one’s language proficiency, language anxiety and WMC, but complexity and fluency may be affected by other factors, and the mechanism involved here is complex [51].

The full model outputs of all regression models are presented in Appendix B.

## 5. Conclusions

This study investigates the main effect of language proficiency, language anxiety and WMC on L3 German language production. Language proficiency is associated with almost all of the outcomes, while WMC and language anxiety are only associated with repetition. Secondly, this study investigates how language proficiency moderates the effects of WMC and language anxiety on language production. Generally, the regression models on most outcomes are significant. As for the WMC × language proficiency interaction, it is found that repetition can be predicted at a significant level and the prediction on repetition is better for low-proficiency speakers. When it comes to the WMC × language anxiety interaction, this study fails to find any predictive power of the interaction on any outcomes. These findings deepen the understanding of how Language proficiency, language anxiety and WMC predict language production, and especially how WMC predicts one’s language production across different Language proficiency levels. This study enriches the language production evaluation dimensions by trying to include meaning construction evaluation in language production tests by adopting a comprehensive FFE model, combining the CAF and CCF models.

At the same time, this study has several limitations. The first limitation of this study is that the self-reported language proficiency may be influenced by the subjects’ language anxiety, which may affect the final result, although a low correlation between language proficiency and language anxiety is reported. Future studies could incorporate more objective language proficiency tasks so as to conduct a more comprehensive evaluation on subjects’ language proficiency [32,33]. Also, the small number of raters cannot ensure high inter-rater reliability, and as a result, several outcomes could not be used in the regression analysis. Most of these outcomes are metric outcomes, and missing these outcomes may result in difficulties differentiating what aspects of the CCF model can be predicted. Moreover, the mixed normalization methods may affect the relative weighting of components, and it is difficult to balance the relative weights of the sub-components in the overall score. Further research could improve the consistency by recruiting more subjects and including more raters. Also, generalizability may be limited because subjects in this study are limited to L1 Cantonese–L3 German college students. These subjects are young and well educated, which ensures that they have sufficient energy and means for language study. However, speakers from different age groups or with different socio-economic status may show a different performance. Hence, more studies along the same lines as explored here, but other participants are called for.

## Figures and Tables

**Table 1:** CAF outcomes.

Complexity			Fluency		Accuracy	
MDD	ED	MATTR	Repetition proportion	Retracing proportion	Word error proportion	Utt. error proportion

MDD = Mean Dependency Distance; ED = Embedding Depth; MATTR = moving average type-token ratio; Utt. = Utterance.

**Table 2:** The CCF model.

Dimensions	Outcomes
Ideational	Overall theme
	Core components
	Background
Textual	Temporality
	Reference
	Connectivity

**Table 3:** The ICC results of the outcomes.

Outcomes	ICC	Inclusion
Overall theme	0.351	FALSE
Core components	0.204	FALSE
Background	0.838	TRUE
Temporality	0.549	FALSE
Reference	0.527	FALSE
Connectivity	0.917	TRUE

**Table 4:** Outcomes: mean values and SDs.

Outcomes	Mean (n = 91)	SD (n = 91)	Outcomes	Mean (n = 91)	SD (n = 91)
WMC	0.636	0.197	Fluency	1.414	0.342
Language proficiency	−0.094	1.006	Word errors	−0.198	0.160
Language anxiety	3.703	0.806	Utterance errors	−0.026	0.018
MDD	4.967	1.995	Accuracy	1.409	0.325
ED	4.575	2.470	Background	4.797	2.781
MATTR	0.591	0.108	Connectivity	6.151	6.524
Complexity	1.783	0.533	CCF	1.011	0.492
Repetition	−0.054	0.048	FFE	5.617	0.948
Retracing	−0.029	0.026	CAF	4.606	0.638

MDD = Mean Dependency Distance; ED = Embedding Depth; MATTR = moving average type-token ratio.

**Table 5:** The correlation coefficients among predictors and outcomes.

	WMC	Pr.	An.	FFE	CAF	Com.	Acc.	Flu.	CCF
WMC	1.00	0.15	0.10	0.14	0.18	0.10	0.10	0.08	0.05
Pr.	0.15	1.00	−0.01	0.05	0.10	0.04	−0.07	0.19	−0.04
An.	0.10	−0.01	1.00	−0.14	−0.07	−0.11	−0.11	0.14	−0.18
FFE	0.14	0.05	−0.14	1.00	0.88	0.65	0.58	0.07	0.79
CAF	0.18	0.10	−0.07	0.88	1.00	0.72	0.51	0.26	0.40
Com.	0.10	0.04	−0.11	0.65	0.72	1.00	0.07	−0.28	0.33
Acc.	0.10	−0.07	−0.11	0.58	0.51	0.07	1.00	−0.09	0.46
Flu.	0.08	0.19	0.14	0.07	0.26	−0.28	−0.09	1.00	−0.20
CCF	0.05	−0.04	−0.18	0.79	0.40	0.33	0.46	−0.20	1.00

WMC = working memory capacity; Pr. = language proficiency; An. = language anxiety; Com. = complexity; Acc. = accuracy; Flu. = fluency.

**Table 6:** Adjusted R^2^ of the language proficiency, anxiety and WMC models on oral production.

	Pr. + WMC + An.	WMC × Pr. + An.	An. × Pr. + WMC	WMC × An. + Pr.
FFE	0.18 *	0.19 *	0.17 *	0.17 *
CCF	0.16 *	0.18 *	0.16 *	0.17 *
Ideational Background	0.12	0.12	0.11	0.11
Textual Connectivity	0.22 *	0.31 *	0.23 *	0.22 *
CAF	0.10 *	0.09 *	0.09 *	0.09 *
complexity	0.02	0.05	0.01	0.01
MATTR	0.03	0.03	0.02	0.03
MDD	0.05	0.06	0.04	0.04
ED	0.00	0.00	0.00	0.01
Accuracy	0.23 *	0.22 *	0.23 *	0.23 *
Word errors	0.24 *	0.23 *	0.23 *	0.24 *
Utterance errors	0.05 *	0.05 *	0.05 *	0.04 *
Fluency	0.02	0.04	0.01	0.01
Retracing	−0.02	−0.03	−0.02	−0.03
Repetition	0.04	0.10 *	0.03	0.03

Pr. = language proficiency; WMC = working memory capacity; An. = language anxiety; MDD = Mean Dependency Distance; ED = Embedding Depth; MATTR = moving average type-token ratio. * refers to the significant outcomes.

**Table 7:** The coefficients of outcomes in different models.

Outcomes	Model	An.	WMC	Pr.	WMC × An.	An. × Pr.	WMC × Pr.
Word errors	Pr. + WMC + An.	0.01	−0.01	0.08 *	-	-	-
	WMC × Pr. + An.	0.01	−0.01	0.08 *	-	-	0.01
	An. × Pr. + WMC	0.00	−0.01	0.08 *	-		-
	An. × WMC + Pr.	0.00	−0.01	0.08 *	0.02	-	-
Utterance errors	Pr. + WMC + An.	0.00	0.00	0.00 *	-	-	-
	WMC × Pr. + An.	0.00	0.00	0.01 *	-	-	0.00
	An. × Pr. + WMC	0.00	0.00	0.00 *	-	0.00	-
	An. × WMC + Pr.	0.00	0.00	0.00 *	0.00	-	-
Accuracy	Pr. + WMC + An.	0.03	−0.01	0.16 *	-	-	-
	WMC × Pr. + An.	0.03	−0.01	0.17 *	-	-	−0.01
	An. × Pr. + WMC	0.03	−0.01	0.16 *	-	0.01	-
	An. × WMC + Pr.	0.02	−0.01	0.17 *	0.03	-	-
Textual Connectivity	Pr. + WMC + An.	−0.38	0.95	2.97 *	-	-	-
	WMC × Pr. + An.	−0.21	0.92	2.04 *	-	-	2.41
	An. × Pr. + WMC	−0.42	0.90	3.05 *	-	−0.71	-
	An. × WMC + Pr.	−0.41	0.98	2.97 *	0.13	-	-
CCF	Pr. + WMC + An.	−0.02	0.03	0.21 *	-	-	-
	WMC × Pr. + An.	−0.01	0.03	0.17 *	-	-	0.09
	An. × Pr. + WMC	−0.02	0.03	0.21 *	-	0.01	-
	An. × WMC + Pr.	−0.03	0.04	0.21 *	0.06	-	-
FFE	Pr. + WMC + An.	−0.03	−0.04	0.43 *	-	-	-
	WMC × Pr. + An.	−0.02	−0.05	0.38 *	-	-	0.15
	An. × Pr. + WMC	−0.03	−0.04	0.44 *	-	−0.02	-
	An. × WMC + Pr.	−0.04	−0.04	0.44 *	0.03	-	-
CAF	Pr. + WMC + An.	−0.02	−0.08	0.23 *	-	-	-
	WMC × Pr. + An.	−0.01	−0.08	0.20 *	-	-	0.06
	An. × Pr. + WMC	−0.02	−0.08	0.23 *	-	−0.03	-
	An. × WMC + Pr.	−0.01	−0.08	0.23 *	−0.02	-	-
Repetition	WMC × Pr. + An.	−0.01 *	0.01 *	0.01	-	-	−0.02 *

Pr. = language proficiency; An. = language anxiety. * refers to the significant outcomes.

**Table 8:** The post hoc interaction test result of “WMC × Pr. + An.” on repetition.

Out	Qua	IC	CI95_i_L	CI95_i_U	I_p	I_p_ BH	Sq.	SE_q	CI95_q_L	CI95_q_U	q_P	q_p_ BH
Repetition	Q25	−0.02 *	−0.02	−0.01	0.00	0.02	0.02	0.01	0.01	0.03	0.00	0.01
Repetition	Q50	−0.02	−0.02	−0.01	0.00	0.02	0.01	0.00	0.00	0.02	0.01	0.08
Repetition	Q75	−0.02	−0.02	−0.01	0.00	0.02	0.00	0.01	−0.01	0.01	0.39	0.67

Out = outcomes; Qua = quartile group; IC = Interaction_Coef; CI95_i_L = CI95_interaction_Lower; CI95_i_U = CI95_interaction_Upperr; I_p = Interaction_p_raw; I_p_BH = Adjusted Interaction_p_BH; Sq. = Slope_quartile; SE_q = SE_quartile; CI95_q_L = CI95_q_lower; CI95_q_U = CI95_q_upper; q_P = *p* value of quartile raw; q_p_BH = adjusted p_quartile_BH. * refers to the significant outcome.

## Data Availability

The original data presented in this study are available in L3HK Repository at https://osf.io/djc69/ (accessed on 1 July 2026), The secondary analysis data is available at https://osf.io/gx3zp/overview (accessed on 1 July 2026).

## Supplementary Materials

The following supporting information can be downloaded at: https://osf.io/gx3zp/overview (accessed on 1 June 2026).

## Appendix A

Handbook of Model Evaluation
1. Ideational
Overall theme: the word “Frosch” appears in a meaningful sentence
4 points—4times; 3 points—2–3 time;
2 points—once; 1 point—implicit
0 point—not mentioned in any way
Core components: Lost Frogs, Find Frogs, Take Frogs Back
2 points—all elements; 1 point—2 elements; 0 point—1elements
Background: Meaningful turns before the frogs are lost
2. Textual
Temporality: (number of verbs—errors in tense)/number of verbs
Reference:
(number of reference expression—errors in reference expression)/ number of reference expression
Connectivity: 0.5 point—one blended sentence 并列举 (make a list)
1 point—one complicated sentence or connected adverbial (Deshalb)

## Appendix B

**Table A1:** The VIF results.

	WMC	Pr.	An.	WMC × Pr	An. × Pr	WMC × An.
Pr. + WMC + An.	1.03	1.02	1.01	-	-	-
WMC × Pr. + An.	1.03	1.25	1.02	1.23	-	-
An. × Pr. + WMC	1.04	1.04	1.01	-	1.02	-
An. × WMC + Pr.	1.07	1.02	1.08	-	-	1.10

Pr. = language proficiency; WMC = working memory capacity; An. = language anxiety.

**Table A2:** The original and adjusted p of the language proficiency, anxiety and WMC models on oral production.

Outcomes	Pr. + WMC + An		WMC × Pr. + An.		An. × Pr. + WMC		WMC × An. + Pr.	
	Raw_p	Adj_*p*	Raw_p	Adj_*p*	Raw_p	Adj_*p*	Raw_p	Adj_*p*
FFE	0.00	0.00	0.00	0.00	0.00	0.00	0.00	0.00
CCF	0.00	0.00	0.00	0.01	0.00	0.01	0.00	0.01
Ideational Background	0.05	0.08	0.04	0.06	0.05	0.08	0.04	0.07
Textual Connectivity	0.01	0.03	0.01	0.02	0.02	0.03	0.01	0.03
CAF	0.01	0.02	0.01	0.02	0.02	0.03	0.02	0.04
Complexity	0.18	0.23	0.04	0.06	0.31	0.33	0.24	0.26
MATTR	0.20	0.23	0.16	0.18	0.23	0.28	0.17	0.21
MDD	0.06	0.09	0.05	0.07	0.11	0.16	0.12	0.17
ED	0.22	0.23	0.17	0.18	0.28	0.32	0.16	0.21
Accuracy	0.00	0.00	0.00	0.00	0.00	0.00	0.00	0.00
Word errors	0.00	0.00	0.00	0.00	0.00	0.00	0.00	0.00
Utterance errors	0.01	0.02	0.01	0.02	0.00	0.01	0.02	0.04
Fluency	0.17	0.23	0.06	0.07	0.18	0.23	0.21	0.25
Retracing	0.80	0.80	0.80	0.80	0.84	0.84	0.90	0.90
Repetition	0.05	0.09	0.00	0.00	0.10	0.16	0.10	0.16

Pr. = language proficiency; WMC = working memory capacity; An. = language anxiety; MDD = Mean Dependency Distance; ED = Embedding Depth; MATTR = moving average type-token ratio; adj_*p* = adjusted *p* value.

**Table A3:** The full model out puts of “Pr. + WMC + An.”.

Outcomes	Pre	Coe	SE	S_b	CI95_L	CI95_U	*p*	Adj_*p*	spR^2^
Word errors	(int.)	−0.20	0.01	NA	−0.23	−0.17	0.00	NA	NA
Word errors	WMC	−0.01	0.01	−0.09	−0.04	0.01	0.26	0.26	0.01
Word errors	Pr.	0.08	0.02	0.52	0.05	0.11	0.00	0.00	0.19
Word errors	An.	0.01	0.01	0.03	−0.02	0.03	0.72	0.72	0.00
Utterance errors	(int.)	−0.03	0.00	NA	−0.03	−0.02	0.00	NA	NA
Utterance errors	WMC	0.00	0.00	0.02	0.00	0.00	0.82	0.82	0.00
Utterance errors	Pr.	0.00	0.00	0.27	0.00	0.01	0.00	0.00	0.08
Utterance errors	An.	0.00	0.00	0.08	0.00	0.00	0.38	0.38	0.01
Accuracy	(int.)	1.41	0.03	NA	1.35	1.47	0.00	NA	NA
Accuracy	WMC	−0.01	0.03	−0.03	−0.07	0.05	0.70	0.70	0.00
Accuracy	Pr.	0.16	0.02	0.51	0.12	0.21	0.00	0.00	0.21
Accuracy	An.	0.03	0.03	0.08	−0.03	0.08	0.36	0.36	0.01
Textual Connectivity	(int.)	6.15	0.60	NA	4.95	7.35	0.00	NA	NA
Textual Connectivity	WMC	0.95	0.68	0.15	−0.39	2.30	0.16	0.16	0.02
Textual Connectivity	Pr.	2.97	0.93	0.45	1.12	4.82	0.00	0.00	0.08
Textual Connectivity	An.	−0.38	0.61	−0.06	−1.58	0.83	0.53	0.53	0.00
CCF	(int.)	1.01	0.05	NA	0.92	1.10	0.00	NA	NA
CCF	WMC	0.03	0.05	0.07	−0.07	0.14	0.51	0.51	0.00
CCF	Pr.	0.21	0.05	0.42	0.11	0.31	0.00	0.00	0.17
CCF	An.	−0.02	0.04	−0.03	−0.11	0.07	0.71	0.71	0.00
FFE	(int.)	5.62	0.09	NA	5.44	5.80	0.00	NA	NA
FFE	WMC	−0.04	0.09	−0.05	−0.22	0.13	0.63	0.63	0.00
FFE	Pr.	0.43	0.09	0.46	0.25	0.62	0.00	0.00	0.21
FFE	An.	−0.03	0.09	−0.03	−0.21	0.15	0.72	0.72	0.00
CAF	(int.)	4.59	0.06	NA	4.47	4.72	0.00	NA	NA
CAF	WMC	−0.08	0.06	−0.12	−0.19	0.04	0.18	0.18	0.01
CAF	Pr.	0.23	0.07	0.35	0.09	0.36	0.00	0.00	0.08
CAF	An.	−0.02	0.06	−0.03	−0.14	0.10	0.79	0.79	0.00

WMC = working memory capacity; Pr. = language proficiency; An. = language anxiety; Pre = predictors; *p* = original *p* value; CI95_L = CI95_Lower; CI95_U = CI95_upper; int. = intercept; Coe = Coefficient; adj_*p* = adjusted *p* value; S_b = Standardized beta coefficients; spR^2^ = semi-partial R^2^. NA = Not Available.

**Table A4:** The full model outputs of “WMC × Pr. + An.”.

Outcomes	Pre	Coe	SE	S_b	CI95_L	CI95_U	*p*	Adj_*p*	spR^2^
Repetition	(int.)	−0.05	0.00	NA	−0.06	−0.04	0.00	NA	NA
Repetition	WMC	0.01	0.00	0.16	0.00	0.01	0.03	0.03	0.03
Repetition	Pr.	0.01	0.01	0.17	0.00	0.02	0.10	0.10	0.02
Repetition	An.	−0.01	0.00	−0.24	−0.02	0.00	0.02	0.02	0.06
Repetition	WMC × Pr.	−0.02	0.00	−0.29	−0.02	−0.01	0.00	0.046	0.07
Word errors	(int.)	−0.20	0.02	NA	−0.23	−0.17	0.00	NA	NA
Word errors	WMC	−0.01	0.01	−0.09	−0.04	0.01	0.25	0.25	0.01
Word errors	Pr.	0.08	0.02	0.49	0.05	0.11	0.00	0.00	0.19
Word errors	An.	0.01	0.01	0.04	−0.02	0.04	0.68	0.68	0.00
Word errors	WMC × Pr.	0.01	0.01	0.06	−0.02	0.04	0.45	1.00	0.00
Utterance errors	(int.)	−0.03	0.00	NA	−0.03	−0.02	0.00	NA	NA
Utterance errors	WMC	0.00	0.00	0.02	0.00	0.00	0.81	0.81	0.00
Utterance errors	Pr.	0.01	0.00	0.30	0.00	0.01	0.00	0.00	0.08
Utterance Errors	An.	0.00	0.00	0.07	0.00	0.00	0.43	0.43	0.01
Utterance errors	WMC × Pr.	0.00	0.00	−0.08	0.00	0.00	0.33	1.00	0.01
Accuracy	(int.)	1.41	0.03	NA	1.35	1.47	0.00	NA	NA
Accuracy	WMC	−0.01	0.03	−0.03	−0.07	0.05	0.71	0.71	0.00
Accuracy	Pr.	0.17	0.03	0.52	0.11	0.23	0.00	0.00	0.21
Accuracy	An.	0.03	0.03	0.08	−0.03	0.08	0.38	0.38	0.01
Accuracy	WMC × Pr.	−0.01	0.03	−0.02	−0.06	0.05	0.75	1.00	0.00
Textual Connectivity	(int.)	5.80	0.49	NA	4.84	6.77	0.00	NA	NA
Textual Connectivity	WMC	0.92	0.58	0.14	−0.23	2.07	0.12	0.12	0.02
Textual Connectivity	Pr.	2.04	0.55	0.31	0.95	3.13	0.00	0.00	0.08
Textual Connectivity	An.	−0.21	0.52	−0.03	−1.25	0.82	0.68	0.68	0.00
Textual Connectivity	WMC × Pr.	2.41	1.26	0.33	−0.10	4.92	0.06	1.00	0.09
CCF	(int.)	1.00	0.05	NA	0.91	1.09	0.00	NA	NA
CCF	WMC	0.03	0.05	0.07	−0.06	0.13	0.49	0.49	0.00
CCF	Pr.	0.17	0.05	0.35	0.07	0.27	0.00	0.00	0.10
CCF	An.	−0.01	0.05	−0.02	−0.10	0.08	0.82	0.82	0.00
CCF	WMC × Pr.	0.09	0.06	NA	−0.03	0.21	0.14	1.00	0.02
FFE	(int.)	5.60	0.09	NA	5.41	5.78	0.00	NA	NA
FFE	WMC	−0.05	0.08	−0.05	−0.21	0.12	0.59	0.59	0.00
FFE	Pr.	0.38	0.11	0.40	0.16	0.59	0.00	0.00	0.13
FFE	An.	−0.02	0.09	−0.02	−0.21	0.16	0.81	0.81	0.00
FFE	WMC × Pr.	0.15	0.10	NA	−0.06	0.35	0.15	1.00	0.02
CAF	(int.)	4.59	0.07	NA	4.46	4.72	0.00	NA	NA
CAF	WMC	−0.08	0.06	−0.12	−0.19	0.04	0.17	0.17	0.01
CAF	Pr.	0.20	0.08	0.32	0.05	0.36	0.01	0.01	0.08
CAF	An.	−0.01	0.06	−0.02	−0.13	0.11	0.85	0.85	0.00
CAF	WMC × Pr.	0.06	0.07	0.08	−0.08	0.20	0.41	1.00	0.01

WMC = working memory capacity; Pr. = language proficiency; An. = language anxiety; Pre = predictors; *p* = original *p* value; CI95_L = CI95_Lower; CI95_U = CI95_upper; int. = intercept; Coe = coefficient; adj_*p* = adjusted *p* value; S_b = standardized beta coefficients; spR^2^ = semi-partial R^2^. NA = Not Available.

**Table A5:** The full model out-puts of “An. × Pr. + WMC”.

Outcomes	Pre	Coe	SE	S_b	CI95_L	CI95_U	*p*	Adj_*p*	spR^2^
Word errors	(int.)	−0.20	0.01	NA	−0.23	−0.17	0.00	NA	NA
Word errors	An.	0.00	0.01	0.03	−0.02	0.03	0.74	0.74	0.00
Word errors	Pr.	0.08	0.01	0.53	0.06	0.11	0.00	0.00	0.19
Word errors	WMC	−0.01	0.01	−0.09	−0.04	0.01	0.23	0.23	0.01
Word errors	An. × Pr.	−0.01	0.01	−0.07	−0.04	0.02	0.43	1.00	0.00
Utterance errors	(int.)	−0.03	0.00	NA	−0.03	−0.02	0.00	NA	NA
Utterance errors	An.	0.00	0.00	0.09	0.00	0.00	0.33	0.33	0.01
Utterance errors	Pr.	0.00	0.00	0.26	0.00	0.01	0.01	0.01	0.08
Utterance errors	WMC	0.00	0.00	0.03	0.00	0.00	0.76	0.76	0.00
Utterance errors	An. × Pr.	0.00	0.00	0.11	0.00	0.00	0.11	1.00	0.00
Accuracy	(int.)	1.41	0.03	NA	1.35	1.47	0.00	NA	NA
Accuracy	An.	0.03	0.03	0.08	−0.03	0.08	0.34	0.34	0.01
Accuracy	Pr.	0.16	0.02	0.50	0.12	0.21	0.00	0.00	0.21
Accuracy	WMC	−0.01	0.03	−0.03	−0.07	0.05	0.73	0.73	0.00
Accuracy	An. × Pr.	0.01	0.02	0.04	−0.03	0.06	0.55	1.00	0.00
Textual Connectivity	(int.)	6.15	0.60	NA	4.96	7.34	0.00	NA	NA
Textual Connectivity	An.	−0.42	0.68	−0.06	−1.77	0.92	0.53	0.53	0.00
Textual Connectivity	Pr.	3.05	0.98	0.47	1.10	5.01	0.00	0.00	0.08
Textual Connectivity	WMC	0.90	0.64	0.14	−0.37	2.16	0.16	0.16	0.02
Textual Connectivity	An. × Pr.	−0.71	0.89	−0.11	−2.49	1.06	0.43	1.00	0.00
CCF	(int.)	1.01	0.05	NA	0.92	1.11	0.00	NA	NA
CCF	An.	−0.02	0.05	−0.03	−0.11	0.07	0.72	0.72	0.00
CCF	Pr.	0.21	0.05	0.42	0.10	0.31	0.00	0.00	0.17
CCF	WMC	0.03	0.05	0.07	−0.07	0.14	0.50	0.50	0.00
CCF	An. × Pr.	0.01	0.04	NA	−0.08	0.10	0.82	1.00	0.00
FFE	(int.)	5.62	0.09	NA	5.44	5.80	0.00	NA	NA
FFE	An.	−0.03	0.09	−0.04	−0.22	0.15	0.71	0.71	0.00
FFE	Pr.	0.44	0.10	0.46	0.25	0.63	0.00	0.00	0.21
FFE	WMC	−0.04	0.09	−0.05	−0.22	0.13	0.62	0.62	0.00
FFE	An. × Pr.	−0.02	0.09	NA	−0.19	0.15	0.79	1.00	0.00
CAF	(int.)	4.59	0.06	NA	4.47	4.72	0.00	NA	NA
CAF	An.	−0.02	0.06	−0.03	−0.14	0.11	0.77	0.77	0.00
CAF	Pr.	0.23	0.07	0.36	0.09	0.37	0.00	0.00	0.08
CAF	WMC	−0.08	0.06	−0.13	−0.20	0.03	0.17	0.17	0.01
CAF	An. × Pr.	−0.03	0.06	−0.05	−0.15	0.09	0.58	1.00	0.00

WMC = working memory capacity; Pr. = language proficiency; An. = language anxiety; Pre = predictors; *p* = original *p* value; CI95_L = CI95_Lower; CI95_U = CI95_upper; int. = intercept; Coe = coefficient; adj_*p* = adjusted *p* value; S_b = Standardized beta coefficients; spR^2^ = semi-partial R^2^. NA = Not Available.

**Table A6:** The full model out-puts of “An. × WMC. + Pr”.

Outcomes	Pre	Coe	SE	S_b	CI95_L	CI95_U	*p*	Adj_*p*	spR^2^
Word errors	(int.)	−0.20	0.01	NA	−0.23	−0.17	0.00	NA	NA
Word errors	An.	0.00	0.01	0.00	−0.03	0.03	0.98	0.98	0.00
Word errors	WMC	−0.01	0.01	−0.02	−0.04	0.02	0.45	0.45	0.01
Word errors	Pr.	0.08	0.01	0.13	0.05	0.11	0.00	0.00	0.19
Word errors	An. × WMC	0.02	0.01	0.03	−0.01	0.05	0.12	1.00	0.00
Utterance errors	(int.)	−0.03	0.00	NA	−0.03	−0.02	0.00	NA	NA
Utterance errors	An.	0.00	0.00	0.00	0.00	0.01	0.46	0.46	0.01
Utterance errors	WMC	0.00	0.00	0.00	0.00	0.00	0.80	0.80	0.00
Utterance errors	Pr.	0.00	0.00	0.01	0.00	0.01	0.00	0.00	0.08
Utterance errors	An. × WMC	0.00	0.00	0.00	0.00	0.00	0.85	1.00	0.00
Accuracy	(int.)	1.41	0.03	NA	1.34	1.47	0.00	NA	NA
Accuracy	An.	0.02	0.03	0.03	−0.04	0.08	0.55	0.55	0.01
Accuracy	WMC	−0.01	0.03	−0.01	−0.07	0.06	0.85	0.85	0.00
Accuracy	Pr.	0.17	0.02	0.26	0.12	0.21	0.00	0.00	0.21
Accuracy	An. × WMC	0.03	0.03	0.04	−0.04	0.10	0.40	1.00	0.00
Textual Connectivity	(int.)	6.14	0.64	NA	4.88	7.40	0.00	NA	NA
Textual Connectivity	An.	−0.41	0.61	−0.64	−1.63	0.81	0.51	0.51	0.00
Textual Connectivity	WMC	0.98	0.69	1.52	−0.39	2.34	0.16	0.16	0.02
Textual Connectivity	Pr.	2.97	0.93	4.63	1.13	4.81	0.00	0.00	0.08
Textual Connectivity	An. × WMC	0.13	0.73	0.19	−1.33	1.59	0.86	1.00	0.00
CCF	(int.)	1.01	0.05	NA	0.91	1.10	0.00	NA	NA
CCF	An.	−0.03	0.05	−0.06	−0.12	0.06	0.52	0.52	0.00
CCF	WMC	0.04	0.06	0.09	−0.07	0.16	0.44	0.44	0.01
CCF	Pr.	0.21	0.05	0.43	0.11	0.31	0.00	0.00	0.18
CCF	An. × WMC	0.06	0.06	NA	−0.05	0.17	0.31	1.00	0.01
FFE	(int.)	5.61	0.09	NA	5.43	5.80	0.00	NA	NA
FFE	An.	−0.04	0.10	−0.04	−0.24	0.16	0.69	0.69	0.00
FFE	WMC	−0.04	0.10	−0.04	−0.24	0.16	0.71	0.71	0.00
FFE	Pr.	0.44	0.09	0.46	0.25	0.62	0.00	0.00	0.21
FFE	An. × WMC	0.03	0.11	NA	−0.19	0.25	0.76	1.00	0.00
CAF	(int.)	4.60	0.07	NA	4.47	4.73	0.00	NA	NA
CAF	An.	−0.01	0.07	−0.02	−0.14	0.12	0.88	0.88	0.00
CAF	WMC	−0.08	0.06	−0.13	−0.21	0.04	0.20	0.20	0.01
CAF	Pr.	0.23	0.07	0.35	0.09	0.36	0.00	0.00	0.08
CAF	An. × WMC	−0.02	0.07	−0.04	−0.16	0.11	0.73	1.00	0.00

WMC = working memory capacity; Pr. = language proficiency; An. = language anxiety; Pre = predictors; *p* = original *p* value; CI95_L = CI95_Lower; CI95_U = CI95_upper; int. = intercept; Coe = coefficient; adj_*p* = adjusted *p* value; S_b = Standardized beta coefficients; spR^2^ = semi-partial R^2^. NA = Not Available.
